# Perspectives on the side effects of hormonal contraceptives among women of reproductive age in Kitwe district of Zambia: a qualitative explorative study

**DOI:** 10.1186/s12905-023-02561-3

**Published:** 2023-08-18

**Authors:** Bright Mukanga, Natasha Mwila, Herbert Tato Nyirenda, Victor Daka

**Affiliations:** Michael Chilufya Sata School of Medicine, Public Health Department, The Copperbelt University, P.O Box 71769, Ndola, Zambia

**Keywords:** Hormonal, Contraceptives, Family Planning, Perspectives on side effects

## Abstract

**Background:**

Globally, hormonal contraceptives have proved to be effective in the prevention of unwanted pregnancies. However, despite evidence of the many benefits associated with the use of hormonal contraceptives, concerns related to their safety and side effects have been reported. We conducted a study to explore the perspectives on the side effects of hormonal contraceptives among women of reproductive age in Kitwe district of Zambia.

**Methods:**

An explorative qualitative study was done among 32 women of reproductive age (18–45 years). Participants were selected conveniently as they accessed family planning services at a designated reproductive, maternal, and child health facility. Data collection was done through in-depth interviews (IDIs). Recruitment of participants and data collection continued until the saturation point was reached. The interviews were recorded, translated, and transcribed verbatim. Data were imported into NVivo.x64 for coding and node generation after which categories and themes were developed manually.

**Results:**

Overall, participants demonstrated a considerable amount of knowledge of family planning, recounting the economic and health benefits as well as demerits of family planning use. The main reasons for discontinuing and switching hormonal contraceptive methods were the desire to get pregnant and the fear of unpleasant side effects, including excessive bleeding or prolonged menstruation, headache, dizziness, lower abdominal/back pain, and weight gain. Most importantly, participants cited concerns about the delay in the resumption of fertility after the termination of contraception and how the side effects disrupted their daily activities at home.

**Conclusion:**

There is a need for family planning providers to offer family planning services that address the side effects of hormonal contraceptives during counselling and how women can manage them. Family planning services should adopt a patient-centred approach that takes into consideration the concerns regarding side effects and how this affects the quality of life among women. Also, there is a need to extend family planning services to include scheduled follow-ups and clinical management of contraceptive side effects among women.

**Supplementary Information:**

The online version contains supplementary material available at 10.1186/s12905-023-02561-3.

## Background information

Contraception or birth control is the intentional prevention of conception or pregnancy through the use of different devices, chemical agents such as spermicides and hormonal contraceptives, drugs, sexual practices, and surgical operations [1]. The various forms of contraceptives include oral contraceptive pills, implants, injectables, patches, vaginal rings, intrauterine devices, condoms, male and female sterilization, lactational amenorrhea methods, withdrawal, and fertility awareness-based methods [2]. Substantial evidence suggests that side effects associated with contraceptive use are a potential deterrent to their consistent use among women [3]. However, consistent use of contraceptives can greatly reduce the risk of unintended pregnancy [4, 5].

Globally, the use of modern contraception had slightly risen from 54% to 1990 to 57.4% in 2015 [6]. Whilst modern methods such as hormonal contraceptives are efficacious in improving the reproductive and sexual health of women as well as reducing maternal and child mortality rates [7], low usage continues to be reported in low-resource settings, with a prevalence of 25% reported in Middle and Western Africa, and 43% in East Africa [8]. Additionally, Mwansa and colleagues reported a 2.5 and 4.6 Total Fertility Rate (TFR) globally and in Sub-Sahara Africa, respectively [9]. Lack of access to contraceptives, male partner influence, lack of education, and negative attitudes by providers have been shown to influence the uptake and utilisation of contraceptives among women [7]. Importantly, perceived and experienced side effects associated with modern contraceptives contribute largely to discontinuation and switching among women [10].

Hormonal contraceptives can prevent conception by stopping ovulation. They contain one or two female sex hormones similar to those naturally produced in a woman’s body. Their use has been a part of clinical practice for more than 50 years [11]. Hormonal contraceptives include methods such as injectables, implants, and oral contraceptives [12]. Despite modern advances in contraceptive methods and evidence of their efficacy, up to 214 million women had unmet needs for family planning in 2017 [10]. Additionally, there are reported alterations in the normal menstrual pattern which is associated with hormonal contraceptive use [13]. Unlike condoms, hormonal contraceptives do not offer protection against sexually transmitted infections (STIs) and can contribute to risk compensation [14]. Concerns over experienced and perceived side effects of hormonal contraception such as menstrual disruption, pelvic cramps, and excessive pelvic bleeding have been highlighted [15]. Also,  the delay in the return of fertility after discontinuation of contraception continues to be a big challenge for most women and poses a negative impact on utilisation of contraception [7, 10, 16]. Further, there is a growing number of literature that have highlighted the influence of culture on women’s perception and utilisation of hormonal contraceptives [17, 18].

In the past decades, Zambia has made steady progress in enhancing knowledge and access to contraceptives among women [19]. This led to a decrease in the fertility rate from 6.75 births per woman in 1955 to 5.5 births per woman in 2015. Despite these gains, Zambia is still among the top 10 countries with high fertility rates, with variations in contraceptive uptake observed between women in rural and urban areas [20]. Many of the studies done on modern contraceptives in Zambia have revealed that the age of the woman, the age of the male partner, residence, educational level, working status, desire for more children, and ethnicity are some of the factors associated with modern contraceptive use [20]. Also, there is still evidence of a high rate of discontinued use of modern contraceptives among women which have been associated with side effects of hormonal contraception [21]. Whilst many studies have addressed the determinants and side effects of modern contraceptives among women, there is a paucity of studies in Zambia that have highlighted the perspectives of women on the side effects of hormonal contraceptives despite theoretical validation of the evidence regarding the discontinued use [22]. Therefore, this study aimed at exploring women’s perspectives on the side effects of hormonal contraceptives.

## Study site

The study was conducted at Ndeke urban clinic located in Kitwe district in the Copperbelt Province of Zambia. The township has three (3) urban clinics. We purposefully chose Ndeke urban clinic as a study site because it has a delivery facility and offers reproductive, maternal, and child health services such as family planning and antenatal services. Ndeke urban clinic covers a catchment area with a population of approximately 100,000 people [23]. The main socio-economic activities in Ndeke township are mining and small business enterprises.

### Sampling and target Population

The target population was women aged between 18 years and 45 years who had come to access family planning services at Ndeke Urban Clinic in Kitwe. We employed convenient sampling to select 32 women aged between 18 and 45 years to participate in 32 in-depth interviews. Participant recruitment into the study continued until data saturation.

### Study design

We employed an explorative qualitative design. The study utilized the naturalistic nature of the explorative research design to gain a deeper understanding of participants’ experiences within their social and natural settings.

### Inclusion and exclusion criteria

We included women aged between 18 and 45 years who came to access family planning services. Only those who consented to participate in the study were included. We excluded those who did not consent and those who were below the age of 18 years or above the age of 45.

### Data collection

Consenting participants were interviewed in a separate room at the clinic facility. Through prolonged engagement with the participants for more than 4 weeks, we conducted face-to-face IDIs in English and Bemba (common local language), which lasted for 50–60 min. We interviewed 32 women as they accessed contraceptives and data only saturated after 4 weeks. The interview guide constituted questions on the contraceptive methods that the women knew; contraceptives used, side effects experienced, and reasons for contraceptive use. All interviews were digitally recorded using a recorder and notes were taken down during the interviews. Interviews continued until the saturation of data and did not proceed thereafter.

### Data analysis

The digitally recorded interviews were transcribed and translated by a qualified language translator. For participants interviewed in Bemba (Local language), we engaged a qualified language translator who translated bemba transcripts into English and back translated them to Bemba for translation accuracy. Content analysis was carried out through code classification and theme identification. All transcripts were reviewed against the recordings for similarity and accuracy [4]. Each researcher had a code book and code books were compared to agree on one single code book. Data were then imported into NVivo.x64 to help in the coding process through the development of nodes. The first (BM) and second (NM) authors reviewed and coded all narratives from the participants and searched for similarities, commonalities, and patterns. We manually developed categories and established themes and subthemes. As recommended by Granehein and Lundman [24] we employed the following steps.

1. Transcribing the interviews verbatim and reading through the text several times to obtain a sense of the whole.
2. Dividing the text into condensed meaning units.
3. Abstracting the condensed meaning units and labelling with codes.
4. Sorting codes into categories and subcategories based on comparing their similarities and differences; and.
5. Formulating themes as the expression of the latent content of the text [25].

### Ethical consideration

Written consent was sought from the Copperbelt University School of Medicine administration. Ethical approval was also obtained from the Tropical Disease and Research Centre (TDRC) (IRB Registration Number: 00002911 & FWA Number: 00003729), and permission was granted by the National Health Research (NHRA) (Ref No: NHRA000011/10/2022). Written informed consent was obtained from the participants before the collection of data. They were informed about the purpose, benefits, and possible harms of the study. Participation was voluntary and those that opted to withdraw were allowed to do so. The information collected from the respondents was kept in a locked cabinet and access to these documents was limited to the researchers only. No identifying information such as names of participants was used as codes were assigned instead.

### Trustworthiness of the study

We employed Guba and Lincoln’s criteria of credibility, transferability, dependability, and confirmability to enhance the trustworthiness of the study findings [26]. Credibility in the study was achieved through prolonged engagement with the participants to understand the context setting of the phenomenon. To achieve this, we spent more than 4 weeks at the health facility engaging the participants and service providers as we recruited and interviewed the participants. This helped us to build trust and rapport with the participants. We also conducted peer debriefing where we invited a qualitative research expert to evaluate and critique our field notes. Further, we invited 2 qualitative research experts to cross-check the data across categories of participants to enhance triangulation. We achieved transferability through the thick description and robust data by employing accurate descriptions of the participants in their natural settings. This was also achieved through continuous recruitment of participants and data collection until data saturation. In this regard, interviewing additional participants was critical in increasing the scope, adequacy, and appropriateness of the data [27]. Dependability was achieved by having our transcript validated by 2 qualitative research experts in reproductive and sexual health. The experts reviewed the themes and the descriptors to note similarities. Confirmability was achieved through an audit trail. This was done by a qualitative research expert who examined the qualitative processes we had taken from data collection, transcription up to interpretation. This was also done by reviewing the documents and transcripts and all the interview notes that we had collected.

## Results

### Participant characteristics

The IDIs were conducted with 32 female respondents ranging from 18 to 45 years of age (Table 1). As all respondents were female, the age range was categorized into 2 groups: 18–35 and 36–45 years of age. The characteristics are tallied with the age ranges, marital status, level of education, and occupation. Most of the respondents were married and between the age range of 18–35 i.e., 36 and 45 respectively. Most of the women interviewed had obtained some form of education, with only 5 not attending school at all.

**Table 1:** Study Participant Characteristics

	Age group (years)	
	**18–35 (22)** **(n=%)**	**36–45 (10)** **(n=%)**
Marital status		
Married	16 (50%)	9(28.1)
Single	6 (18.7%)	1(3.1%)
Divorced	0	0
Widowed	0	0
Level of education		
Junior Secondary Level (G8-G9)	3 (9.3%)	0
No Formal Education	4 (12.5%)	1 (3.1%)
Primary Level (G1-G7)	3(9.3%)	2(6.2%)
Senior Secondary Level (G10-G12)	6(18.7%)	1(3.1%)
Tertiary Level	6(18.7%)	6 (18.7%)
Occupation		
Accountant	1(3.1)	0
Banker	0	1 (3.1%)
Businesswoman	3 (9.3%)	5 (15.6%)
Farmer	3(9.3%)	0
Health worker	1(3.1%)	4(12.5%)
Housewife	10(31.2%)	0
Student	1 (3.1%)	0
Teacher	3(9.3%)	0

### Theme 1: contraceptive knowledge

#### Methods known

Most participants were knowledgeable about the contraceptive methods available namely, pills, injectables, implants, and intra-uterine devices. The most common method known by the women were pills, injectables, and implants.

*“The methods that I know are pills, loop, jadelle, and injections…” (27-year-old, currently using the injection)*.

#### Benefits of family planning

Despite inherent side effects associated with contraceptive use, some participants held positive views of modern contraceptive methods and their benefits. The most cited benefit was the ability to space the children through the prevention of pregnancy. One of the participants, a 34-year-old who had an implant had this to say.

*“If you’re not using family planning, you’ll be having children without any child spacing…… it helps you to plan for your future and your children’s future” (34-year-old, currently using an implant)*.

#### Demerits of contraceptive use

However, some participants had concerns regarding prolonged menstruation and how it affected their quality of life and their health.

*“Family planning is very important because it helps you space up your children, however, it also has negatives such as having prolonged menses and affecting one’s blood pressure” (37-year-old, currently on the pill)*.

### Perceptions of attributed side effects

We noted variations in the perceived side effects of hormonal contraceptives among women. However, all hormonal contraceptives were generally linked to menstrual irregularity, prolonged bleeding, cramping or stomach pain, and percieved infertility. Women also outlined headaches, dizziness, irregular periods, weight gain, and weight loss as side effects of hormonal contraceptives. Perceptions of the attributed hormonal contraceptive side effects varied among the respondents. Respondents mainly expressed concerns about how the side effects would affect their normal day-to-day activities and disrupt their health or well-being. Some participants linked the side effects to the delay in the resumption of fertility.

## Themes regarding perceived side effects of hormonal contraceptives

### Theme 2: perceived side effects (excessive bleeding, cramps, headaches)

Participants cited side effects which ranged from pain, headaches, weight gain, and prolonged bleeding. Some participants linked the attributed side effects to other health conditions as depicted in the excerpt below.

*“I continued to bleed after insertion of the implant. I went to the clinic to have it removed but I was told it couldn’t be done as it was just inserted. I later became anaemic and it was removed” (34-year-old implant user).*

Some women recounted the side effect associated with hormonal contraceptive use such as cramps, dizziness, weight gain, and headaches.

*“I experienced, dizziness, weight gain, cramps like the ones I would get when I am going to start my period. I would also have headaches. These would stop after some time and I did not face challenges getting tasks of the day done” (43-year-old, previous injectable user)*.

Because of associated side effects such as headaches and waist pain, some participants resorted to discontinuation and switching contraceptives.

*“I used to take the pill called Safe Plan but I feared missing the time. I changed to another pill called Microgynon but I would get headaches. So I started getting the three-month injection and I would have cases where my period would start from nowhere plus I went for 3 months without my period. I then tried the intrauterine device but got terrible waist pain. I had it removed and then went back to the pill (Oralcon) but it run out of stock. I didn’t take it for a while and I became pregnant (34-year-old current implant user).*

### Theme 3: hindrance to performance of normal daily duties

Participants recounted how the attributed side effects affected their ability to perform daily domestic activities at home such as cooking and caring for the children as depicted in the following excerpt by a 43-year-old woman who had previously used injectables.

*“I experienced some cramps like the ones I would get when I am going to start my period. I would also have headaches. These would stop after some time and I did not face challenges getting tasks of the day done” (43-year-old, previous injectable user)*.

Similarly, A 38-year-old, mother of 2 who had an implant narrated how she could fall sick the entire day and would require resting whenever pain could kick in.

*“I would feel strong pulls like those that I experience when I am about to start my period, but I would not bleed. I would feel sick the entire day and require some time to sit or rest whenever the pain kicks in” (38- year-old, mother of 2, currently with an implant).*

### Theme 4: delay in the resumption of fertility after contraception discontinuation

Most participants linked the side effects of contraceptives to a delay in conceiving after discontinuation of contraception. Specifically, participants narrated how their menstruation cycles became irregular after they had stopped using hormonal contraceptives. Participants narrated how it became difficult for them to conceive after they had stopped using hormonal contraceptives.

*“The challenge I can say I had with the 3-year implant was taking long to conceive after its removal. 1 year passed before I got pregnant” (28-year-old mother of 2, currently not using any method of contraception)*.

## Discussion

This study explored the perspectives of women of reproductive age on the side effects of hormonal contraceptives in Kitwe District of Zambia. For women to make informed decisions regarding contraceptive use, a certain amount of knowledge of contraceptive methods is a requirement [28]. In the current study, we found that most women were knowledgeable about various methods of hormonal contraceptives. The most common methods cited were implants, injectables, pills, and IUDs (Intrauterine Devices). Other studies done elsewhere have shown similar results where high knowledge regarding hormonal contraceptives was recorded [10, 29]. In the current study, all married respondents were able to name more than one method of contraception and had used or were using at least one contraceptive method. Twenty-two (22) out of the 32 participants (1/3) had a considerably good knowledge of methods of contraception which is consistent with the findings of a study in Ethiopia [30]. Studies elsewhere have reported low levels of knowledge of modern contraceptives among women [31, 32]. The regional variations in knowledge regarding modern contraceptives could be due to differences in attributable variables such as post-primary education, ethnicity, residence, and discussion of family planning with a spouse [33, 34].

Women’s experiences of unfavorable side effects accord critical constraints to contraceptive usage [3, 7, 35]. Common side effects associated with the use of hormonal contraception include headaches, weight gain, depression, irregular menstrual bleeding, and fatigue [7]. Also, study findings elsewhere have revealed an association between hormonal contraceptives with an increased risk of venous thrombosis, ischemic stroke, and heart attack in some women [36–38]. However, this needs to be understood in the context of other risk factors such as smoking, age, personal history of thrombosis, inherited thrombophilia and lifestyle choices [37]. While the benefits of hormonal contraceptives generally outweigh the risks, women need to be educated and aware of these potential risks so that they discuss them with their healthcare providers when accessing hormonal contraceptives.

In the current study, the main side effects highlighted by participants were headaches, lower abdominal/ back pain, cramps, excessive or prolonged bleeding, and delay in the resumption of menstruation. Among these, the most common side effect after discontinuation was prolonged and irregular menstrual bleeding. Similar findings have been reported in Uganda where hormonal contraceptives were associated with severe bleeding during and after discontinuation [3]. However, we note that variabilities exist in how women respond and perceive contraceptive-induced menstrual bleeding which could be shaped by individual and social influences [39]. We suggest that when providing family planning counselling, healthcare providers should explore the unique needs of each woman and understand how prolonged menstrual bleeding affects their quality of life and how bleeding can be managed.

Concerns regarding excessive menstrual bleeding, menstrual changes, and associated abdominal cramps prevented women from conducting daily routine work at home. In this regard, excessive pelvic bleeding associated with hormonal contraceptives may present a practical challenge to menstrual hygiene management in some women, particularly those of low social economic status [3, 40–42]. Wealth-related inequality in menstrual hygiene has been documented. Specifically, prolonged bleeding poses a challenge to women of low social economic status who struggle to access sanitary pads, have private, clean, and safe spaces for practicing menstrual hygiene[43].

Most women had perceived misconceptions about side effects associated with hormonal contraceptive use such as prolonged menstrual bleeding and vagina dryness. This has been reported elsewhere [16]. Misconceptions about contraceptive side effects could be due to insufficient information on the role of contraceptives on the human reproductive system, peer influence from male partners, and other close relations [7]. For instance, in the current study, most women did not know how hormonal contraceptives influence the menstrual cycle and the role of menstruation in reproduction. This can affect women’s choice and utilization of hormonal contraception. Apart from the need for providers to discuss the role of hormonal contraception on menstrual suppression in women [44], there is a need for improved family planning counselling before and during the use of contraceptives [45–47]. When women are well informed about the role of contraceptives in pregnancy prevention and anticipated side effects, they are likely to make informed decisions regarding the choice of contraceptives.

As above, perceived and experienced side effects of hormonal contraceptives can influence women’s perceptions of hormonal contraceptives. This has the potential to affect women’s decisions to commence, change and continue using contraceptives [7, 48]. Despite high knowledge of methods of hormonal contraceptives among women, fear and misconceptions about side effects was a dividing gap between knowledge and attitudes towards the uptake of contraceptives which led to the discontinuation of contraception use. Previous studies have shown that this is the case in most low-income countries [4, 7, 35]. Given this, comprehensive and accurate information about family planning methods and side effects and how they can be managed needs to be incorporated into routine family planning counselling programmes [4, 35, 49]. This can improve uptake and enhance the continued use of contraceptives among women [50]. Importantly, family planning providers should not only aim at giving out contraceptives and explaining the associated side effects but there should be a deliberately scheduled follow-up plan and clinical management of contraceptive side effects [35].

Delayed return to a normal menstrual cycle after discontinuation of hormonal contraceptives is a major concern and a common barrier to contraceptive use among women [51]. In the current study, one of the major concerns was the delay in the resumption of the menstruation cycle after the termination of contraception. Similar findings have been reported elsewhere [17, 52]. However, to date, there is inconclusive evidence on the effect of parity in the resumption of pregnancy after contraceptive discontinuation [16]. We suggest that besides giving assurance to women to use their preferred contraceptive methods through counselling, there is a need for a patient-centred approach that takes into consideration how side effects affect the quality of life and how they can be managed [53].

Our study was not without limitations. As is the case in qualitative research, we notice that the findings of the study could be limited in their applicability to a marginalized segment of the population due to inherent selection bias. This is because some women could have decided not to come to the health facility and access contraception due to fear of known contraception side effects. However, the explorative nature of the qualitative study enabled a thick description of the phenomenon under study within the participant’s natural setting.

## Conclusion

Concerns regarding side effects such as prolonged menstrual bleeding were highlighted by most participants. Further, participants recounted how side effects affected their daily routine work and house chores. Besides influencing the uptake and contributing to a high discontinuation rate of hormonal contraceptive use, side effects can present a practical challenge to menstrual hygiene management among women. Therefore, family planning services should adopt a patient-centred approach that takes into consideration the concerns regarding side effects and how this affects the quality of life among women. There is a need to extend family planning services to scheduled follow-up, and clinical management of contraceptive side effects.

### Electronic supplementary material

Below is the link to the electronic supplementary material.

Supplementary Material 1

## Data Availability

The datasets analyzed during this study are available from the corresponding author on reasonable request.

## Declarations

### Statement

All methods were performed in accordance with relevant guidelines and regulations.
